# 

**Published:** 1893-01-21

**Authors:** 


**Notices of Births, Deaths, and Marriages,** are inserted in this column at a charge of 2s. 6d. for an announcement not exceeding four lines.

### NOTICE TO CORRESPONDENTS.

All MS., Letters, Books for Review, and other matters intended for the Editor should be addressed THE EDITOR, THE LODGE, PORCHESTER SQUARE, LONDON, W.

The Editor cannot undertake to return rejected M.S., even when accompanied by stamped directed envelope.

**Notice to Advertisers and Subscribers.**

All Advertisements, Orders for Copies of THE HOSPITAL, and Business Communications should be addressed to The Publisher, *not to the Editor*, THE HOSPITAL, 140, Strand, W.C.

The Cost of Subscription, post free, is as follows:—Per week, 2½d.; per quarter, 2s. 6d.; per half-year, 5s.; per annum, 8s. 6d. Foreign:—Per annum, 10s. 8d.; payable, in all cases, in advance.

**"Burdett's Hospital Annual," 1893.**

Fourth year of publication. 700 pp. Boards, gilt lettering. A most complete guide to British, Colonial, Indian, and American Hospitals, Dispensaries, Nursing and Convalescent Institutions, and Asylums. Price 5s. Published by THE SCIENTIFIC PRESS (Limited), 140, Strand, W.C.

**NOTICE.—The Index for Vol. XII. (ending September 1892) is on sale at the Office of this Journal, price 2½d., post free.**

### SCRAPS AND GLEANINGS.

THREE fresh cases of cholera have been reported from Hamburg.

IN Paris the number of hospital beds has risen from 8,152 in 1872 to 12,486 in 1893.

A COTTAGE HOME has recently been opened at Clacton-on-Sea for Waifs and Strays.

THE Queen sent some toys for the Christmas treat to the Victoria Hospital for Children.

THE Queen has sent 60 lb. of old linen for the use of the patients in the London Hospital.

EIGHT HUNDRED AND EIGHTEEN POUNDS has been collected at Hull in aid of the Sunday Hospital Fund.

THE new Hospital for Infectious Diseases, Gorse Hill, near Swindon, has been erected at a cost of £4,080 by Mr. G. Wiltshire.

PRINCESS MAY has collected £200 towards clearing off a debt still remaining on the Victoria Hospital for Invalid Children at Margate.

THE new ward at the Richmond Hospital is, with the gracious consent of Princess Victoria Mary of Teck, to be known as the "Princess Mary's Ward."

THE Infectious and Hospital Sub-Committee, Croydon, have, with the Medical Officer, decided upon the plans for the proposed new infectious hospital.

IT is now proved that freezing does not purify water. The average amount of impurity retained by the ice is 34·3 per cent. of organic matter, and 21·2 per cent. of inorganic.

THE Lord Provost of Edinburgh suggests that a Royal Commission should inquire into the subject of the education of tinkers' children, of which there are some 2,000 in Scotland.

A SILVER MEDAL has been awarded to Mr. W. Pestell, coxswain of the Palling lifeboats, in recognition of services extending over 22 years, in which period the boats have saved 398 lives.

HOT-WATER fountains are already in Paris. This is a great benefit to the working poor, who by an ingenious method can turn on eight litres of hot water by dropping one sou into the slot.

SMALL-POX is reported from several centres in Durham. The sanitary authorities are doing all they can to prevent the disease from spreading by isolating the sufferers wherever possible.

THE proposal to erect a public hospital at Falmouth has been met with considerable support. A substantial fund being already promised for the building fund, it only remains to choose a suitable site.

BY direction of the Princess of Wales, £25 18s. 6d. has been forwarded to the Secretary of the British Home for Incurables, this being a further donation from the proceeds of Canon Fleming's sermon.

THE Governor of Warsaw has lately issued a decree prohibiting the official use of the Polish language in Russian Poland, doctors and nurses in hospitals being forbidden to converse to patients in that language.

IT is estimated that there is a yearly expenditure by well-to-do Americans of 125,000,000 dollars on the support of charitable institutions, and that 500,000,000 dollars are invested in permanent buildings.

THE British Home for Incurables is making an earnest appeal for 500 new subscribers towards expenses incurred in building new premises at Streatham. In addition to inmates, 300 pensions receive £20 annually.

THE new "Cornelia Wing" of the Royal Victoria Hospital, Bournemouth, was opened on the 19th inst. by the Duke of Connaught, who afterwards planted a tree in the grounds. The wing is named after Lady Wimborne.

DR. COLLINGRIDGE, in his report to the Sanitary Committee of London, says he fears there is no doubt of the return of the cholera epidemic with the spring, and that the precautions already existing will be severely tested.

THE inhabitants of Blackpool are bestirring themselves about the dire need of a hospital in that town; indeed, a place that can boast of its Eiffel tower, its three piers, and innumerable new buildings, ought to add an infirmary to its list.

EXTRA accommodation has been made at the Children's Hip Disease Hospital, Sevenoaks. The Lady Superintendent is earnestly begging help towards starting a Cot Endowment Fund. All cases of bone disease requiring a long time of rest are admissible.

A LONDON Board school has lately been deprived of £20 of the usual Government grant on account of the dirt of ceilings and walls. It may be a somewhat severe lesson, but is none the less a salutary reminder to this establishment in Bethnal Green that cleanliness is next to godliness.

THE heart of the French Dauphin, son of Louis XVI. and Marie Antoinette, was removed of the autopsy ninety-seven years ago, since when it has been preserved in alcohol. The son of the physician who performed the autopsy having recently died, the specimen is to be sold by auction.

ALDERMAN TRELOAR recently organised the Guildhall entertainment for 2,000 school children of Farringdon Without. The entertainment consisted of a shadow pantomime, a Christmas tree lighted by electricity, and a tea. The Lord and Lady Mayoress received them afterwards in the Library.

THE one hundred and thirty-second half-yearly Court of Governors of the British Orphan Asylum was held recently. The chairman congratulated the subscribers upon the present satisfactory state of the institution, observing that it was in a sound condition financially, educationally, and morally.

AT a special meeting of the Corporation of the Aberdeen Royal Infirmary and Asylum, it was proposed to reconstruct the old asylum. The Jubilee Fund, amounting to £30,000, with the interest and more has been spent on the new infirmary buildings. The electric light and steam machinery alone cost £6,000.

A PUBLIC meeting will be held at the Mansion House on the 25th inst., at three o'clock, to consider the scheme for providing epileptic persons of both sexes with suitable employment. The Lord Chancellor, Sir Andrew Clarke, Sir James Crichton Browne, Professor Ferrier, and others are announced to address the meeting.

For Student of Medicine and Medical Appointments see pages VI. and VII. overleaf.

### THE STUDENT OF MEDICINE.

[All communications for this Supplement should be addressed to THE EDITOR of THE HOSPITAL, at 140, Strand, with the words, "Students' Column," written in the left hand top corner of the envelope.]

### APPOINTMENTS.

Alexander, S., M.D., B.Ch., B.A.O., appointed *pro tem.* Surgeon to the Belfast Union Infirmary. Allen, Richard Grammar, L.R.C.P.Edin., M.R.C.S.Eng., reappointed Medical Officer of Health for Belper. Bigger, E. C., M.D., M.Ch., L.M., L.R.C.P. Irel., appointed Visiting Medical Officer to the Belfast Union Infirmary. Booth, Frederick, M.B., C.M.Aberd., reappointed Medical Officer of Health for St. Anne's-on-the-Sea Urban Sanitary District. Cartwright, E. H., M.B., B.Ch.Oxon., L.R.C.P.Lond., M.R.C.S., appointed Assistant House Physician to Guy's Hospital. Collins, Henry Beale, M.R.C.S., D.P.H., L.S.A., appointed Medical Officer of Health for Kingston-on-Thames Urban District. Cullin, Richard Blair, L.R.C.P., L.R.C.S.Edin., reappointed Medical Officer of Health for the Borough of Tiverton. Curtis, Albert, M.R.C.S., appointed Medical Officer of Health for the First Sanitary District of the Staines Union. Day, Francis W. H. L., L.R.C.P.Lond., M.R.C.S.Eng., appointed Medical Officer of Health to the Baldock Local Board. Dukes, T. Archibald, M.R.C.S.Eng., L.R.C.P.Lond., appointed House Surgeon to the Croydon General Hospital. Elliott, A. C., M.B., C.M.Edin., appointed Senior House Surgeon at the Preston and County of Lancaster Royal Infirmary. Emtage, Edmund Walter, M.R.C.S.Eng., L.S.A., appointed Medical Officer for the Buckland District of the Bideford Union. Fisher, W. H., M.B., B.C.Camb., appointed House-Physician to Guy's Hospital. Hall, Robert, L.R.C.P., L.M., L.R.C.S.Edin., appointed Visiting Medical Officer to the Belfast Union Infirmary. Hamilton, E. T. E., B.Sc.Lond., F.R.C.S., L.R.C.P., M.R.C.S., appointed Assistant House Surgeon to Guy's Hospital. Harlock, Harry, M.R.C.S., L.R.C.P., reappointed Medical Officer for the Third District of the Westbourne Union. Haswell, William Cyril, M.B., B.S. Durh., appointed House Surgeon to the Royal Infirmary, Newcastle-upon-Tyne. Martin, Charles Lister, M.R.C.S., L.R.C.P. Lond., appointed Medical Officer for the Third District of the Chelmsford Union. Menzies, J., L.R.C.P., L.M., L.R.C.S.Edin., L.F.P.S.Glas., appointed Medical Officer of Health to the Shireoaks Sick and Accident Club, Worksop. Metcalfe, G. E. G., L.R.C.S., L.R.C.P., L.F.P.S.Glas., L.M., appointed Medical Officer for the Capel District of the Tonbridge Union. Monckton, William, L.R.C.P.Edin., M.R.C.S.Eng., appointed Medical Officer of Health to the Portishead Local Board. Napier, Francis, H., M.B., B.S.,Lond., M.R.C.S.Eng., appointed Surgeon to the Glasgow Ophthalmic Institution. Odell, Robert, L.R.C.P.Lond., M.R.C.S.Eng., reappointed Medical Officer for the Second and Third Districts of the Hertford Union. Pimblett, W. H., M.B., C.M.Edin., appointed Junior House Surgeon at the Preston and County of Lancaster Royal Infirmary. Sheen, A. W., M.B., L.R.C.P.Lond., M.R.C.S., appointed House Surgeon to Guy's Hospital. Sichel, G., M.R.C.S., L.R.C.P.Lond., appointed House Surgeon to Guy's Hospital. Southey, Albert James, M.R.C.S.Eng., L.S.A., re-appointed Medical Officer and Public Vaccinator of the Colnbrook District of the Eton Union. Stephens, C., appointed Medical Officer for the Fourth District of the Brockley Union.

### VACANCIES.

The following vacancies are announced this week, the amount of salary in each case being given after the name of the institution:

### Resident Medical Officers.

Chichester Infirmary, Chichester. House Surgeon. Salary £80 per annum, with board, lodging, and washing. Applications to E. E. Street, Secretary, by March 1st.

County Asylum, Prestwich, Manchester, Pathologist. Salary £200 per annum, with furnished apartments, board, attendance, and washing. Applications to the Superintendent.

Derbyshire Royal Infirmary. Assistant House Surgeon. Appointment for six months, but eligible for re-election for additional six months. Salary, £10 for first six months, and £25 for second six months, with separate apartments, board, and washing. Applications to the Secretary by January 19th.

### The Hospital Advertiser.

### MEDICAL APPOINTMENTS.

### APPOINTMENT OF MEDICAL SUPERINTENDENT.

Norfolk House, Norfolk Street, Strand, W.C.,

24th December, 1892. (1,621)

### EAST LONDON HOSPITAL FOR CHILDREN,
Glamis Road, Shadwell, E.

HOUSE SURGEON.

### VICTORIA HOSPITAL, FOLKESTONE.

### CHICHESTER INFIRMARY.

HOUSE SURGEON WANTED.

Salary £80 per annum, with Board, Lodging, and Washing.

### THE STUDENT OF MEDICINE—(*continued*).

Earlswood Asylum for Idiots, Redhill, Surrey. Medical Superintendent; age 30 to 45. Salary £500 per annum, with furnished apartments in the building, coal, and gas. Applications, endorsed "Medical Superintendent," to the Board of Management at the Offices, 36, King William Street, London Bridge, E.C., by January 21st.

East London Hospital for Sick Children, Glamis Road, Shadwell, E. House Surgeon. Board and lodging provided. Applications to the Secretary by January 25th.

General Hospital, Birmingham. Two Assistant House Surgeons. Appointments for six months. Board, residence, and washing provided. Applications to the House Governor by January 28th.

Great Northern Central Hospital, Holloway Road, N. House Surgeon. Salary £60 per annum, with board and lodging. Applications to the Secretary by January 24th.

Guest Hospital, Dudley. Resident Medical Officer. Doubly qualified. Salary, £100 per annum, with board, residence, attendance, and washing. Applications to the Secretary by January 20th.

Metropolitan Asylums Board, Norfolk House, Norfolk Street, Strand, W.C. Medical Superintendent at the Darenth School for Imbeciles, near Dartford, Kent. Doubly qualified. Not more than 45 years of age. Salary, £450 per annum, with furnished residence, coals, gas, milk, garden produce, and washing. Applications, on forms to be obtained at the Office of the Board, to be sent in by January 23rd.

Morpeth Dispensary. House Surgeon. Unmarried. Salary, £120 per annum, with furnished apartments, coal, and gas. Applications to George O. Wright, Honorary Secretary, Beechfield, Morpeth, by January 21st.

North Staffordshire Infirmary and Eye Hospital, Hartshill, Stoke-on-Trent. House Surgeon; doubly qualified. Salary £120 per annum, increasing £10 yearly, with furnished apartments, board, and washing. Applications to the Secretary by January 21st.

Royal South Hants Infirmary, Southampton. Assistant House Surgeon. Board and rooms provided. Applications to T. A. Fisher Hall, Secretary, by January 30th.

Victoria Hospital, Folkestone. House Surgeon, unmarried. Salary £80 the first year, with an increase of £10 each year for the two following years. Applications to the Secretary by January 21st.

West Herts Infirmary, Hemel Hempstead. House Surgeon and Dispenser, doubly qualified, unmarried. Salary £100 per annum, with board, furnished rooms, light, fire, attendance, and washing. Applications to the Secretary by January 31st.

### Non-Resident Medical Officers.

Chester Royal Infirmary. Visiting Surgeon. Doubly qualified. Salary £80 per annum, with residence and maintenance in the house. Applications to the Chairman of the Board by January 21st.

Corporation of Norwich. Medical Officer of Health. Salary, £400 per annum. Applications, endorsed "Medical Officer of Health," to Geo. B. Kennett, Town Clerk, Guildhall, Norwich, by January 28th.

Stratford-on-Avon Rural Sanitary Authority. Medical Officer of Health. Salary £110 per annum. Applications to John Charles Warden, 9, Guild Street, Stratford-on-Avon, by January 21st.

University College of South Wales and Monmouthshire, Cardiff. A Professor of Anatomy, and a Professor of Physiology. Stipend in each case £350 per annum. Applications to Ivor James, Registrar by February 10th.

### University of St. Andrews.

DRAFT MEDICAL ORDINANCES.—The University Commissioners have issued ordinances relating to the allocation of chairs between St. Andrews and Dundee, the formation of faculties and fee funds and the establishment of the chairs necessary to complete the conjoint University School of Medicine. The main provisions of the medical ordinances are similar to those of the other Scottish Universities. The previous ordinance is repealed, and the degree of M.D. conferred on practitioners over 40 years of age is therefore to be abolished. The new chairs to be established are those of pathology and materia medica, medicine, surgery, and midwifery. The Dundee Royal Infirmary is recognised as the official hospital. The arrangements for the constitution of the conjoint medical school are not yet complete, no provision having yet been made for the formation of a fee fund in the Medical Faculty, as in the case of the faculties of Arts and Science. In these faculties the Commissioners ordain the establishment of a common "fee fund" for the chairs in both St. Andrews and Dundee, as well as a "salaries fund," out of which latter a specified minimum salary (£400 to £500) is to be paid. Hereafter the common fee fund (into which the whole of the fees are to be paid) is to be divided proportionately among the occupants of the constitution chairs, to bring the gross salary as far as possible up to a specified "annual" figure, which varies from £500 to £700 in different cases. The matter of pensions is also dealt with. The total sum to be allocated to St. Andrews University is £10,800 per annum, or about one-seventh of the whole amount disbursed to the Scottish Universities. The objects upon which this sum is to be expended are clearly specified, and include the endowments of the new medical chairs, and the equipment of the conjoint medical school. With regard to the medical curriculum, the first year of study (chemistry, physics, zoology, botany) is duplicated, and may be taken either at St. Andrews or Dundee. The courses for the remaining four years are to be provided exclusively in Dundee, and comprise the chairs of anatomy and physiology in University College, along with the new chairs to be instituted, and clinical instruction in the Dundee Royal Infirmary, &c. The endowment for the chair of medicine in St. Andrews is, on the death or resignation of the present occupant, to be diverted to the foundation of a chair in the Arts Faculty in St. Andrews.

